# Safety and feasibility of the transoral endoscopic thyroidectomy vestibular approach with neuroprotection techniques for papillary thyroid carcinoma

**DOI:** 10.1186/s12893-022-01707-8

**Published:** 2022-07-13

**Authors:** Zhen-Xin Chen, Ya-Min Song, Jing-Bao Chen, Xiao-Bo Zhang, Feng-Shun Pang, Zhan-Hong Lin, Li-Ming Yang, Bei-Yuan Cai, You Qin

**Affiliations:** Department of Minimally Invasive Surgery, The Second Affiliated Hospital of Guangzhou University of Chinese Medicine (Guangdong Provincial Hospital of TCM), Guangzhou, 510120 People’s Republic of China

**Keywords:** TOETVA, PTC, Neuroprotection techniques, Safety, Feasibility

## Abstract

**Background:**

This study aimed to evaluate the feasibility and safety of the trans-oral endoscopic thyroidectomy vestibular approach (TOETVA) with neuroprotection techniques for the surgical management of papillary thyroid carcinoma (PTC).

**Methods:**

Patients with PTC who underwent TOETVA between December 2016 and July 2020 were included in this study, and their relevant clinical characteristics, operational details, and surgical outcomes were reviewed and extracted from their medical records for further analysis.

**Results:**

A total of 75 patients successfully underwent TOETVA with zero conversions. Unilateral lobectomy with isthmectomy and total thyroidectomy were completed for 58 and 17 patients, respectively, all using our unique neuroprotective procedure and ipsilateral central neck dissection (CND). The mean number of retrieved lymph nodes versus positive lymph nodes was 6.8 ± 3.7 vs. 1.5 ± 2.3. Postoperative complications included three cases of transient superior laryngeal nerve (SLN) palsy (4.0%), five cases of transient recurrent laryngeal nerve (RLN) palsy (6.7%), 14 cases of transient hypoparathyroidism (18.7%), two cases of numb chin (2.7%) and two cases of flap perforation (2.7%). The follow-up period for patients with PTC lasted for 15.6 ± 10.9 months, during which no other complications or tumor recurrence were observed.

**Conclusion:**

TOETVA can be safely performed for patients with PTC with satisfactory results during the short-term follow-up period. Our neuroprotection techniques can be integrated into TOETVA, which is worth recommending for PTC patients who desire better cosmetic surgical outcomes.

## Introduction

The incidence rate of thyroid carcinoma has been increasing in recent years and the disease ranks fifth in malignancies among women [1]. Thyroidectomy with or without neck lymph node dissection is the preferred treatment for thyroid carcinoma. However, one of the biggest complaints about thyroidectomy, especially from female patients, is the significant scar along the lower neck. Thus, efforts to develop a better operational approach with better cosmetic outcomes remain active.

Since endoscopic surgery for the thyroid was first reported by Huscher et al. in 1997 [2], several cosmesis-driven approaches have been applied in thyroidectomies to avoid neck scarring, including axillary, breast, and retroauricular approaches [3–5]. However, these remote-access approaches still leave scars and require extensive flap dissection before the thyroidectomy. More recently, with the zeal for natural origin transluminal endoscopic surgery [6], the first transoral endoscopic thyroidectomy was performed in living pigs in Germany in 2008 [7]. The technique was subsequently offered to patients as a trans-oral endoscopic thyroidectomy vestibular approach (TOETVA) procedure for thyroid disease with fewer complications. Since then, TOETVA has been highlighted as a non-scar minimally invasive surgery (MIS) among patients and surgeons because of no scar and less trauma [8].

Different groups have reported that compared to traditional surgery, TOETVA results in an insignificant difference in procedure-related complications but achieves outstanding cosmesis [9–12]. However, the procedure is limited to the treatment of benign thyroid tumors or Graves’ disease in early practice [13–16]. Although a few sporadic studies on the application of TOETVA in papillary thyroid carcinoma (PTC) can be found in the literature [10, 11, 17–22], these studies included a relatively small number of patients. Thus, the promotion of TOETVA for the treatment of PTC requires more reliable data from more centers and practical methods to reduce complications.

As one of the most dreadful complications related to thyroid surgery, nerve injury seriously harms patients and reduces their quality of life. During the TOETVA procedure, the narrow operating space and difficulty exposing the superior thyroid increase the likelihood of nerve damage, which raises an urgent need for a standard method for neuroprotection during the procedure. Therefore, we designed the present study to evaluate the safety and feasibility of TOETVA for the treatment of patients with PTC and to provide information regarding our neuroprotection techniques during this novel thyroid resection procedure.

## Materials and methods

### Patients

The clinical data for 75 patients with PTC who underwent TOETVA at our hospital from December 2016 to July 2020 were retrieved from the medical records of the patients and retrospectively assessed. The study protocol was approved by our Institutional Review Board for ethics. The inclusion criteria were as follows: (1) PTC was confirmed by postoperative pathology; and (2) the patient underwent TOETVA willingly. The exclusion criteria were as follows: (1) evidence of distant metastasis; (2) lateral lymph nodes metastasis; (3) invasion of the surrounding tissues; (4) patient previously had surgery or radiation on the neck; (5) patient could not tolerate anesthesia or surgery; and (6) body mass index (BMI) greater than 40 kg/m^2^.

### Preoperative preparation for TOETVA

Patients were admitted to our hospital the day before surgery. Neck ultrasound and CT scans were performed preoperatively to evaluate the tumor in the thyroid gland and the neck lymph node status for the patient. Besides, for suspicious nodules ≤ 5 mm, we routinely recommend observation. But if the patients with nodules ≤ 5 mm (TI-RADS 4b/4c/5) strongly request surgery, TOETVA will be performed without FNA. For suspicious nodules > 5 mm, FNA is recommended to assist in preoperative diagnosis. Gargle was provided to the patients the day before surgery, and prophylactic antibiotics (1st generation cephalosporin, Cefazolin sodium 2 g) were administered 30 min before surgery. As for the selection of appropriate operation, lobectomy and isthmectomy is the recommended initial surgical approach for cT1-2N0M0 PTC patients. Total thyroidectomy may be preferred in cT3 patients or in patients with bilateral malignant nodules. When surgery is considered for patients in either of the following cases: (1) cN1a patients; (2) multiple malignant nodules in unilateral lobe; (3) malignant nodules in one lobe and indeterminate nodules in the contralateral lobe; (4) malignant nodule is adjacent to the trachea, the approach may be modified based on patient preference and patients who avoid the possibility of requiring a future surgery on the contralateral lobe prefer to undergo total thyroidectomy.

### Surgical procedures

**Fig. 1:**
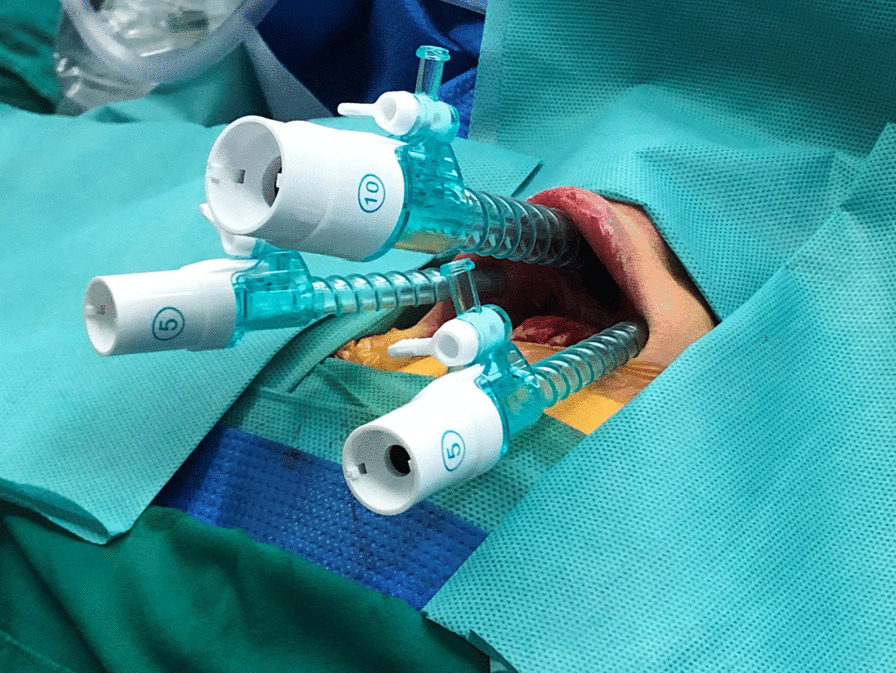
Trocar placement for TOETVA

**Fig. 2:**
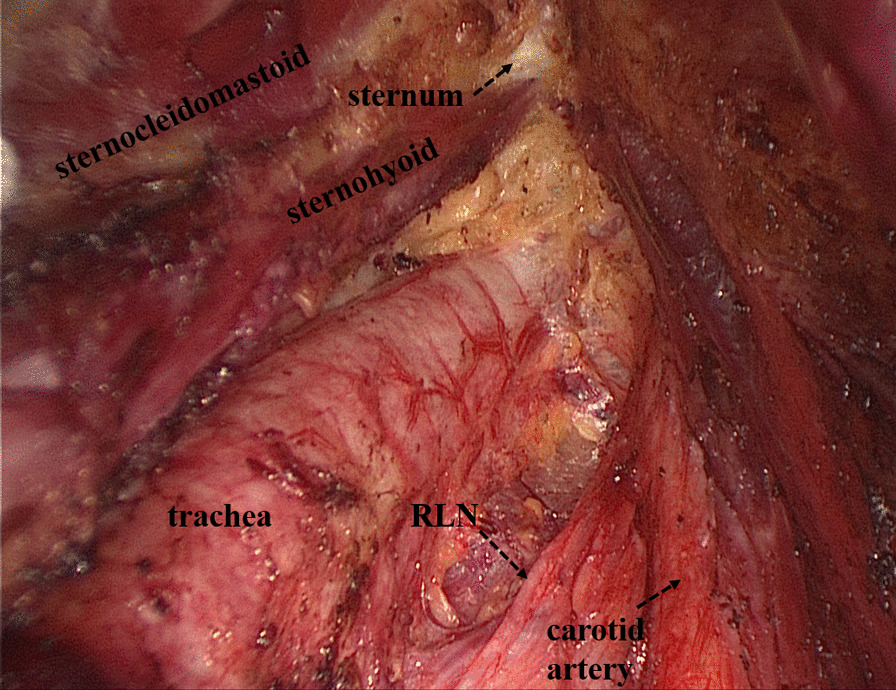
Boundaries of operating space for central neck dissection

The TOETVA surgical standard technique was previously described by Wang and Anuwong [13, 16, 23, 24]. Three trocars (a 10-mm trocar at midline and two 5-mm trocars at the level of the first premolars) were inserted under the lower lip in the oral vestibular area (Fig. 1). After the operating space was created, the linea alba cervicalis was divided to expose the thyroid for dissection and resection. Following thyroidectomy, central neck dissection (CND) was performed with inferior dissection to the brachiocephalic trunk artery and lateral dissection to the carotid artery [25] (Fig. 2).

**Fig. 3:**
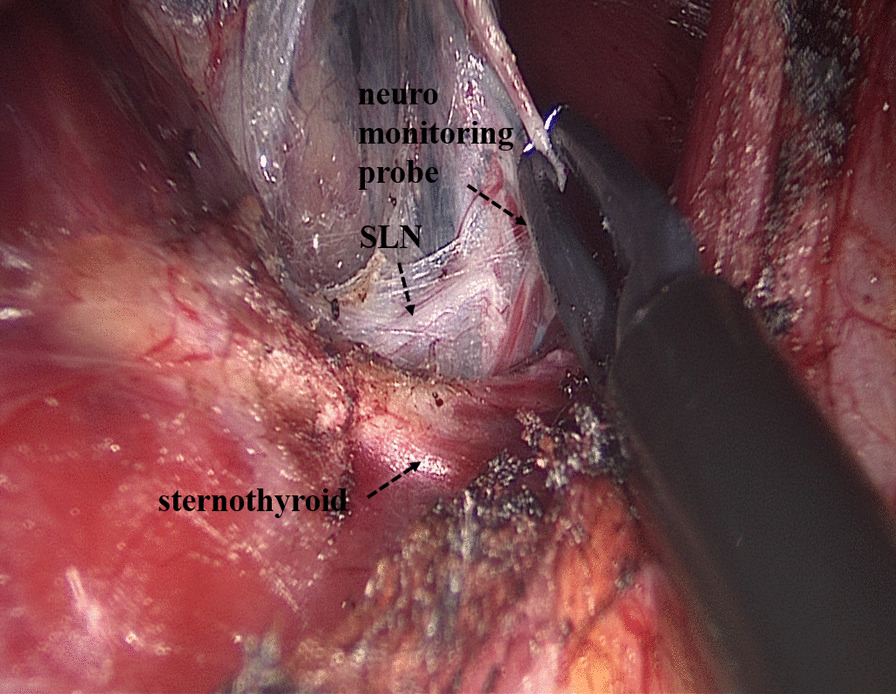
Amputation of sternothyroid to expose superior laryngeal nerve

**Fig. 4:**
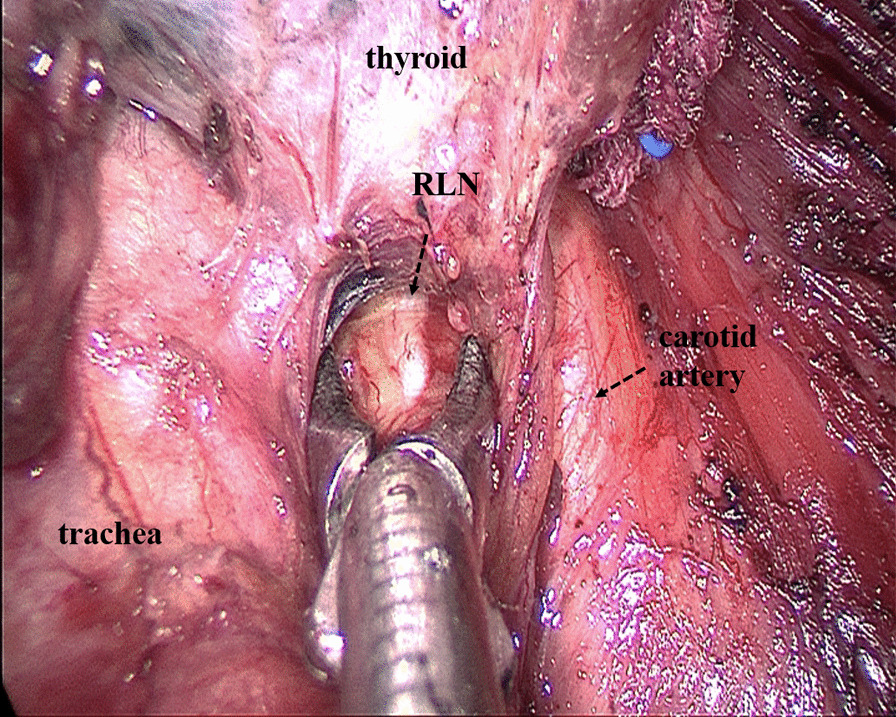
Using "tunnel" exploration at the larynx entry point to protect the recurrent laryngeal nerve

**Fig. 5:**
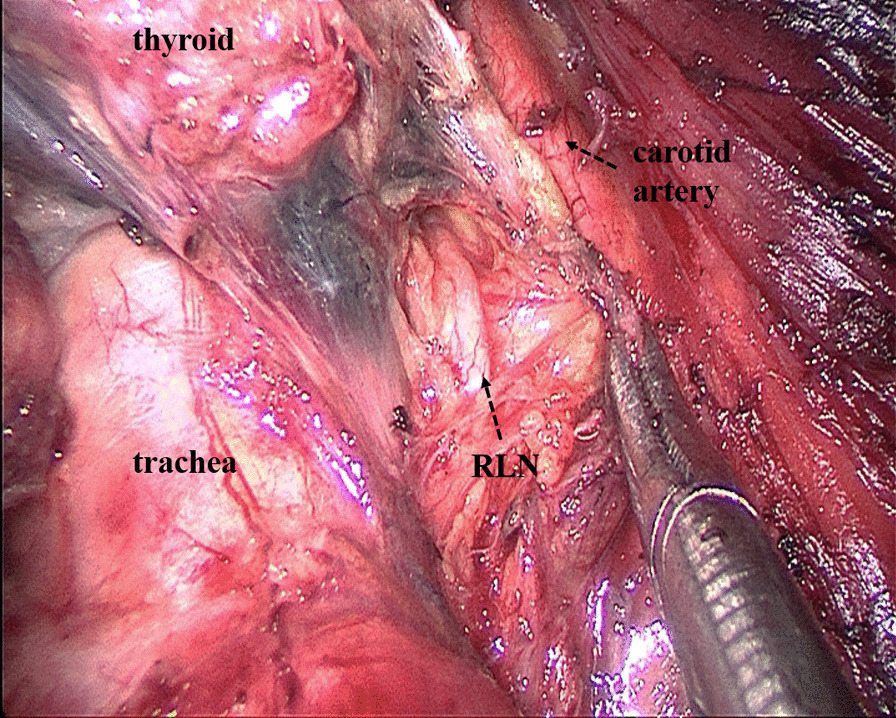
Exposing the recurrent laryngeal nerve from top to bottom

**Fig. 6:**
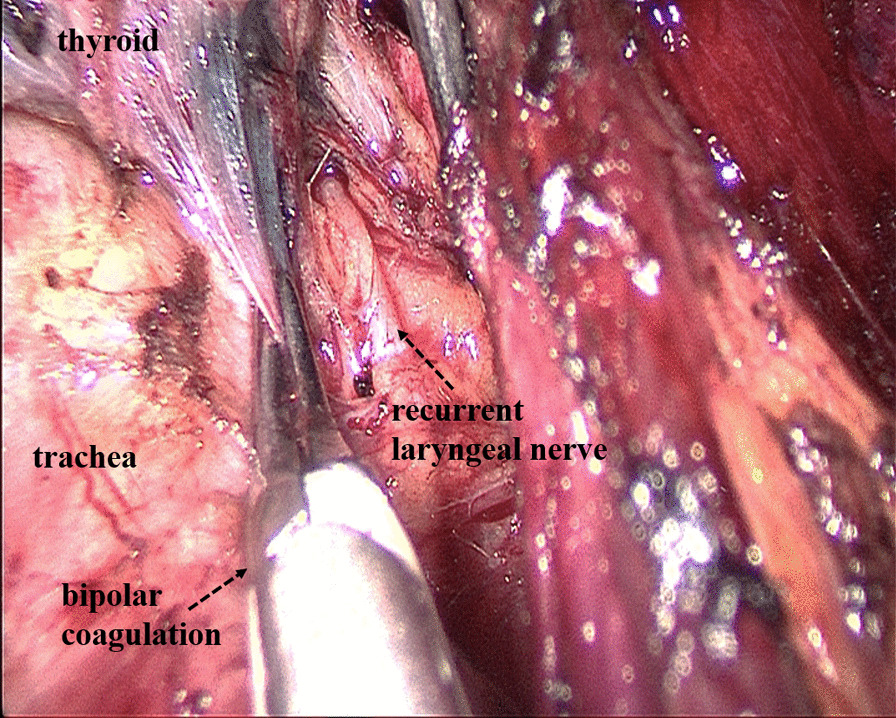
Using bipolar coagulation to resect the thyroid at the larynx entry point

Some operational insights for neuroprotection during TOETVA are as follows: Firstly, enough operation space should be created using the sternocleidomastoid as the landmark for the lateral edge and the sternum as the landmark for the inferior edge (Fig. 2). Secondly, before cutting off the superior pole of the thyroid, part of the sternothyroid should be routinely amputated to increase the visibility of the superior pole of the thyroid, and a nerve monitor should be used to monitor and protect the superior laryngeal nerve (SLN) (Fig. 3). To protect the recurrent laryngeal nerve (RLN), besides the use of a nerve monitor, we also recommend "tunnel" exploration at the larynx entry point using separating pliers (Figs. 4, 5). In addition, a bipolar coagulation device is recommended for the resection of the thyroid at the larynx entry point, which may completely remove the thyroid and protect the RLN (Fig. 6).

### Postoperative management and follow-up

A single additional intravenous dose of antibiotic was administered after surgery (1st generation cephalosporin, Cefazolin sodium 2 g) and the patients return to a normal diet 6 h after surgery. The drainage tubes were removed when the post-operative drainage volume was less than 30 mL/day and the patient was discharged a day later. Hoarseness, low voice, vocal cord function, parathyroid hormone (PTH) concentration, and other complications were assessed or measured as indications of the safety outcomes of TOETVA. The total number of lymph nodes removed, the number of positive central lymph nodes, and tumor recurrence were used as indicators for the feasibility and effectiveness of TOETVA. In this study, hypoparathyroidism was defined when the level of PTH was lower than the lower limit (11 pg/mL) of the normal range of the hormone [4]. Intraoperative neuromonitoring was used in all 75 PTC patients and the SLN damage was evaluated through cricothyroid tremor and EMG during surgery. After surgery, SLN was assessed based on symptoms. Patients were recommended to undergo laryngoscopy to confirm the diagnosis in either of the following cases: (1) disappearance of cricothyroid tremor during surgery; (2) with symptoms of low-pitched voice and the inability to achieve high pitch tasks after surgery. Besides, the patients were assessed for vocal cord function and diagnosed with RLN palsy by laryngoscope. Patients with abnormal EMG signal (the EMG amplitude was decreased by 50%) or postoperative voice impairment were recommended for laryngoscopy to confirm the diagnose. For patients with SLN palsy or RLN palsy, we usually recommend intravenous drip dexamethasone (10mg qd) for 3 days and oral neurotrophic drugs (mecobalamin 0.5mg tid) for a month. For patients with hypoparathyroidism, we recommend the use of calcium tablets, combined with calcitriol if necessary, to maintain normal calcium level [17, 32, 37]. If the injured nerve and parathyroid gland did not recover to preoperative normality after more than half a year, the injury was considered permanent. Patients were re-examined to assess their thyroid hormone levels, TSH at 1 month, 3 months, 6 months, 1 year and annually thereafter. Besides, patients were evaluated by neck ultrasound at 6 months, 1 year and annually thereafter, and the normal neck ultrasound suggested that no regional lymph nodes or recurrent lesions were found. Once suspicious lesion or lymph nodes were detected, FNA or reevaluation of neck ultrasound 3 months later would be recommended. For patients undergone total thyroidectomy, we evaluated additionally by unstimulated-Tg and anti-Tg, and for patients treated by RAI after total thyroidectomy, we evaluated additionally by unstimulated-Tg, anti-Tg and Whole-body Iodine-131 Imaging.

## Results

A total of 75 patients were included in this study and their detailed information is shown in Table 1. All 75 cases were overseen by one surgeon. The surgeon also performed TOETVA on a small number of patients with other diseases, but the data for these patients were incomplete and were not included in the statistics.

**Table 1:** Demographic characteristics of patients with PTC who underwent TOETVA

Variables	Value
Age	36.8 ± 10.5 (20–65)
Sex (female/male)	66/9
BMI	22.3 ± 3.5 (15.8–34.3)
BMI ≥ 25	13
Operation type, n (%)	
Total thyroidectomy with CND	17 (22.7)
Lobectomy with CND	58 (77.3)
Tumor size (cm)	0.83 ± 0.66 (0.1–3.5)
Postoperative stage, n (%)	
T1	69 (92.0)
T2	3 (4.0)
T3b	3 (4.0)
Hospital stay (days)	3.8 ± 1.1 (2–8)
Drainage time (days)	2.8 ± 1.1 (1–7)
Blood loss (mL)	21.1 ± 17.3 (5–100)
Operation time (min)	140.1 ± 48.4 (65–295)
Total thyroidectomy with CND	165.1 ± 44.0 (70–237)
Lobectomy with CND	132.7 ± 47.5 (65–295)
Retrieved lymph nodes	6.8 ± 3.9 (0–17)
Positive lymph nodes	1.5 ± 2.3 (0–11)

*PTC* papillary thyroid carcinoma, *TOETVA* trans-oral endoscopic thyroidectomy vestibular approach, *CND* central neck dissection

Of 75 patients with PTC who underwent TOETVA, 66 patients were women and only nine were men. The mean age of the patients was 36.8 ± 10.5 years, with an age range from 20 to 65 years old. All patients successfully underwent TOETVA with zero conversions to open thyroidectomy. Moreover, 58 patients had unilateral lobectomy plus isthmectomy, and total thyroidectomies were performed for 17 patients. Ipsilateral CND was performed routinely.

**Fig. 7:**
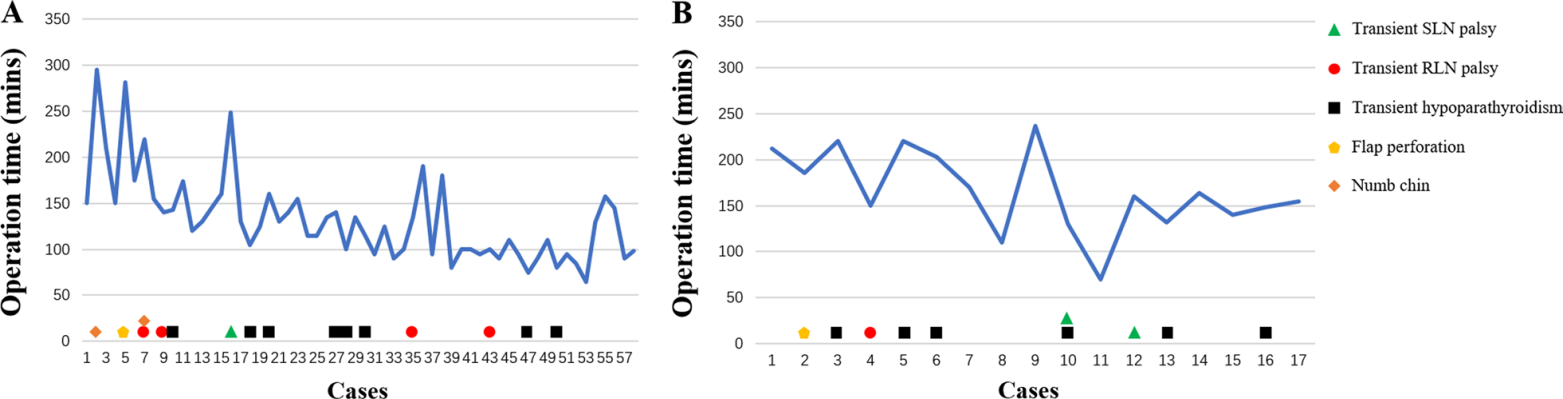
Operating time (mins) and postoperative complications of each patient. **A** 58 patients underwent lobectomy. **B** 17 patients underwent total thyroidectomy

Intraoperatively, the size of the resected tumor was 0.83 ± 0.66 cm with 6.8 ± 3.9 retrieved lymph nodes and 1.5 ± 2.3 positive lymph nodes. The mean operating time for total thyroidectomy and lobectomy was 165.1 ± 44.0 min and 132.7 ± 47.5 min, respectively. The operating time for TOETVA for individual patients plotted in chronological sequence is shown in Fig. 7 as an indicator for the learning course. Blood loss ranged from 5 to 100 mL, with a mean volume of 21.1 mL.

Postoperatively, the mean length of hospital stay after the operation was 3.8 ± 1.1 days with a range of 2 to 8 days (Table 1). Transient SLN palsy and transient RLN palsy were found in three patients (4.0%) and five patients (6.7%), respectively. Fourteen patients (18.7%) had transient hypoparathyroidism, two patients (2.7%) had numb chin and two patients (2.7%) had flap perforation. There was no case of permanent RLN palsy, permanent SLN palsy, permanent hypoparathyroidism or permanent mental nerve injury. Moreover, none of the patients had surgical site infection, aeroembolism, lip tearing or secondary operation due to postoperative events. The mean follow-up period for the patients was 15.6 ± 10.9 months. During the follow-up visits, no other complications or tumor recurrence were found, and the mean Tg of patients undergone total thyroidectomy was 0.93 ± 1.30 ng/ml while the mean anti-Tg was 67.4 ± 85.2 ng/ml half a year after surgery. (Table 2).

**Table 2:** Postoperative complications of patients with PTC who underwent TOETVA

Variables	Value
Transient superior laryngeal nerve palsy	3 (4%)
Transient recurrent laryngeal nerve palsy	5 (6.7%)
Transient hypoparathyroidism	14 (18.7%)
Flap perforation	2 (2.7%)
Numb chin	2 (2.7%)
Permanent superior laryngeal nerve palsy	0
Permanent recurrent laryngeal nerve palsy	0
Permanent hypoparathyroidism	0
Permanent mental nerve injury	0
Surgical site infection	0
Aeroembolism	0
Lip tearing	0
Postoperative bleeding	0
Neck ultrasound	
Normal	75
Abnormal	0
Tg^a^	0.93 ± 1.30 (0.04–4.98)
Anti-Tg^a^	67.4 ± 85.2 (15–291.49)
Other complications^b^	0

*PTC* papillary thyroid carcinoma, *TOETVA* trans-oral endoscopic thyroidectomy vestibular approach, *Tg* Thyroglobulin

^a^For patients undergone total thyroidectomy with CND, we evaluated by Tg and anti-Tg

^b^Including carotid artery injury, chylous fistula, Horner syndrome, hematoma, and tracheal injury

## Discussion

Endoscopic thyroidectomy (ESTC) has evolved steadily over the last 20 years, and the improvements in cosmesis and quality of life make it a popular choice for young patients. With the emergence of TOETVA, the cosmetic effects of thyroid surgery have been further optimized and the disadvantages of large trauma in ESTC have been minimized. However, the promotion of TOETVA for the treatment of PTC requires more clinical data and operational experience sharing. In this study, we evaluated the safety and feasibility of TOETVA for PTC.

Regarding the safety of TOETVA, the main concern for surgeons is the protection of nerves. To better protect the nerves during the dissection of the thyroid gland, some technical points need to be addressed. During TOETVA, difficulties are routinely encountered in exposing the superior pole of the thyroid, increasing the probability of SLN injury. Partial sternothyroid amputation does not normally affect the quality of life of patients but is conducive to the successful exposure and protection of the SLN. Additionally, the RLN is usually located at the larynx entry point; therefore, "tunnel" exploration is recommended at the larynx entry point through the use of separating pliers for quick and accurate identification of the RLN. Furthermore, the thyroid closely approximates the RLN at the larynx entry point; thus, bipolar electrocoagulation is recommended for complete resection of the thyroid at the larynx entry point and to protect the RLN. Using these neuroprotection techniques, transient SLN palsy and transient RLN palsy only occurred in three (4.0%) and five patients (6.7%) in this study, respectively and permanent nerve injury did not occur. Although the probability of temporary nerve injury in this study was similar to that in some reports on open surgery [26–30], we thought that the postoperative RLN palsy is high. We first performed TOETVA in 2016. In the first 14 cases of TOETVA, lack of experience led to the high complication rate of nerve injuries. In the latter 61 cases, only two cases of temporary RLN injury occurred. The neuroprotection techniques introduced in this study are also a summary of our experience accumulated in 75 cases of TOETVA. Therefore, we believe that after accumulating experience, the rate of RLN palsy will gradually decrease. As for the feasibility of TOETVA, the number of lymph nodes removed with TOETVA in this study was 6.8 ± 3.9, which was no less than that in open surgery [10]. As another parameter, the operating time for TOETVA, which was reported in previous studies to be significantly longer than that in open surgery [9], was used to judge the feasibility of the procedure. In this study, although the average operating time for total thyroidectomy and lobectomy were 165.1 ± 44.0 min and 132.7 ± 47.5 min respectively, the time gradually decreased with consecutive TOETVA operations (Fig. 7), demonstrating that the operating time can be reduced with the improvement of surgical proficiency through more experience. Based on the above, we believe that the safety and short-term clinical outcomes for TOETVA are comparable to those of open surgery.

The safety and feasibility of TOETVA have also been confirmed in different centers [31–33]. Compared with previous studies, the rate of permanent RLN injury in this study is lower [18, 20, 34, 35], which may benefit from the neuroprotective techniques mentioned above. However, the complication rate of temporary RLN injury and temporary hypoparathyroidism in our center is higher than that reported in previous studies, and the reasons can be summarized as follows: Firstly, different from other studies that included benign thyroid diseases [16, 24, 34], all included patients in this study diagnosed with PTC. Second, for PTC patients, the mean number of harvested lymph node was higher than that in previous studies [19, 20, 32]. To reduce tumor recurrence, some dangerous operations during thyroidectomy and CND may lead to the increase of the complication rate of temporary RLN injury and temporary hypoparathyroidism. Fortunately, all patients recovered within half a year. In addition, the rate of transient numb chin in this study was 2.7%, which was similar to that reported in other centers [19, 32]. Complications such as tracheal injury [32], Horner syndrome [35, 36] and chylous fistula [35] reported in other studies did not occur in our center.

In this study, the average tumor size removed with TOETVA was only 0.83 ± 0.66 cm, mainly because TOETVA was offered to patients with tumors smaller than 2 cm during the preoperative imaging workup. Four patients who had tumors larger than 2 cm in this study demanded TOETVA despite dissuasion from the surgeon. Factually, the tumor size determined with postoperative pathology is often smaller than that determined with preoperative ultrasound in our center. In addition, the rate of patients needed RAI after total thyroidectomy was 23.5% in this study, which was lower than that in another research [37]. We conclude that this is associated with the low rate of T3b patients in this report (Table 1).

There are several caveats related to the TOETVA procedure. Firstly, the operating space for TOETVA is quite narrow at the beginning of the operation and may suitably accommodate the electric hook due to its small size and flexibility. We believe that reasonable use of an electric hook, especially the electric cutting function, will help expedite the process of initial space establishment in TOETVA. Secondly, carbon nanoparticles can be injected into the inferior pole of the thyroid in order to solve the problem of finding the inferior parathyroid gland. Thirdly, because of the surgical difficulties and complications, TOETVA should be performed by a surgeon with extensive experience in ESTC. Beginners should strictly follow the standard operating procedures and be familiar with neuroprotection techniques. Despite the fact that our surgeon had completed more than 1000 cases of ESTC over a period of more than 10 years, the mean operating time of TOETVA for total thyroidectomy and lobectomy were 165.1 ± 44.0 min and 132.7 ± 47.5 min respectively, which is still much longer than that of open surgery.

The limitations of the study are as follows: First, the clinical data for the patients who underwent open surgery were not included in this study as a comparable reference to improve the level of evidence. Secondly, the mean follow-up period for patients with PTC was only 15.6 ± 10.9 months, which is too short to draw conclusions regarding the effectiveness of TOETVA in the treatment of PTC.

In summary, similar to previous findings, we suggested that the safety and short-term clinical outcomes for TOETVA are comparable to those of open surgery. Moreover, the neuroprotection techniques described in this paper may be helpful to reduce the incidence of permanent RLN injury. In addition, it should be noted that SLN protection techniques and data of SLN injury in TOETVA rarely be presented before. In this study, we shared the neuroprotective skills and experience summarized from 75 cases of TOETVA for PTC. We hope that documentation of our approach and the lessons learnt can facilitate mutual learning and communication, and promote the application of TOETVA in the treatment of PTC.

## Data Availability

The datasets used and/or analysed during the current study are available from the corresponding author on reasonable request.

## Abbreviations

TOETVA: Transoral endoscopic thyroidectomy vestibular approach
PTC: Papillary thyroid carcinoma
CND: Central neck dissection
ESTC: Endoscopic thyroidectomy
RLN: Recurrent laryngeal nerve
SLN: Superior laryngeal nerve
PTH: Parathyroid hormone
